# Ankle-Brachial index by oscillometry: A very useful method to assess peripheral arterial disease in diabetes

**DOI:** 10.4103/0973-3930.62600

**Published:** 2010

**Authors:** M. Premanath, M. Raghunath

**Affiliations:** Prem Health Care, 671, Nrupatunga Road, M-Block, Kuvempunagar, Mysore - 570 023, India; 1 Laxmi Scanning and Color Doppler Center, Dhanvantari Road, Mysore - 570 001, India

**Keywords:** Ankle-Brachial Index, oscillometric method, PAD, risk factors, sensitivity, specificity

## Abstract

**Background:**

Peripheral Arterial Disease (PAD) remains the least recognized form of atherosclerosis. The Ankle-Brachial Index (ABI) has emerged as one of the potent markers of diffuse atherosclerosis, cardiovascular (CV) risk, and overall survival in general public, especially in diabetics. The important reason for the lack of early diagnosis is the non-availability of a test that is easy to perform and less expensive, with no training required.

**Objectives:**

To evaluate the osillometric method of performing ABI with regard to its usefulness in detecting PAD cases and to correlate the signs and symptoms with ABI.

**Materials and Methods:**

Two hundred diabetics of varying duration attending the clinic for a period of eight months, from August 2006 to April 2007, were evaluated for signs, symptoms, and risk factors. ABI was performed using the oscillometric method. The positives were confirmed by Doppler evaluation. An equal number of age- and sex-matched controls, which were ABI negative, were also assessed by Doppler. Sensitivity and Specificity were determined.

**Results:**

There were 120 males and 80 females. Twelve males (10%) and six females (7.5%) were ABI positive. On Doppler, eleven males (91.5%) and three females (50%) were true positives. There were six false negatives from the controls (three each). The Sensitivity was 70% and Specificity was 75%. Symptoms and signs correlated well with ABI positives. Hypertension was the most important risk factor.

**Conclusions:**

In spite of the limitations, the oscillometric method of performing ABI is a simple procedure, easy to perform, does not require training and can be performed as an outpatient procedure not only by doctors, but also by the paramedical staff to detect more PAD cases.

## Introduction

Peripheral Arterial Disease (PAD) is the involvement of arteries of the lower limbs due to atherosclerosis; the detection and appropriate measurement of which is of great importance not only in terms of the disease, but also for the strong predictive power it has for subsequent CV mortality.[1] The mortality due to c*oronary artery disease* (CAD) is two to three times greater in the general population when it is associated with PAD. This holds true even after revascularization.[3] Patients with PAD are at triple the risk of All Cause Mortality (ACM) and at six times the risk of death from Coronary Heart Disease (CHD) as those without the disease.[28,5] PAD is prevalent in 20% of the medical population, but diagnosed in < 50% of the patients with the disease.[10] Even in asymptomatic patients, PAD is a marker of systemic vascular disease involving coronary, cerebral, and renal vessels.[12]

Although much is known about PAD in the general population, the assessment and management of it in diabetes is less clear. It is often more subtle in its presentation with diabetes than without. A classic history of claudication is less common due to peripheral neuropathy.[12] It tends to involve multiple sites of distal vessels of the leg rather than those in the proximal arteries.[2] As the symptoms reduce, the patients do not present for medical evaluation until they have developed medical complications.[2] Eight percent (8%) of the diabetics would have PAD at the time of diagnosis of diabetes, which increases to 45% by 20 years of the duration of the disease.[1]

Despite the importance of the early detection of the atherosclerotic disease, the diagnosis of PAD is often overlooked during routine physical examination. PAD history is elicited in only 37% of the cases, peripheral pulses are palpated in < 60% of the cases, and ABI is performed in < 8% of the cases.[28] To the primary care physician, the medical history and physical examination are the major tools for suspecting and establishing the diagnosis of PAD, but both lack the sensitivity.[19,22]

The ABI helps to define the severity of the disease and a successful screening of the hemodynamically significant disease.[5] The lower the ABI, the greater is the incidence of CV risk factors and clinical CV disease.[4,26] An abnormal ABI in an asymptomatic patient helps in detecting the reservoir of asymptomatic diseases among older people.[3] In spite of all these, PAD remains the least recognized and treated entity by primary care physicians and CV physicians. The office-based assessment of PAD is limited by the need of specialized equipment (handheld Doppler), its cost (rupees 15 – 20 thousand), the time required for performing the test (at least 20 minutes), and the skill of the performer.[1,10]

A test that is automated, easy to perform, and less reliant on specialized skills, may facilitate the performing of ABI in more number of people in less time, thus increasing the diagnosis of PAD in a susceptible population.[10] The present study utilizes an automated digital BP apparatus to measure the ABI in diabetics and attempts to answer the usefulness of this method in detecting an additional number of PAD cases. It also tries to emphasize the importance a clinical examination as well as the assessment of risk factors in the diagnosis of PAD in diabetes.

## Materials and Methods

The enrollment for this study began in August 2006. Patients with diabetes attending the clinic and having the disease for five years or more, irrespective of their symptoms, were included. A few patients, having diabetes for less than five years were also included, to know whether PAD was prevalent in the early years itself. Those who were critically ill or who had severe limb ischemia were not included. Enrollment was stopped in April 2007 when 200 diabetics were included. Informed consent was taken from each patient. Ethical clearance was also obtained from the ethical committee of the trust that sponsored this study. A proforma that was prepared for this study was used to maintain uniformity. A detailed history of diabetes, hypertension, smoking, dyslipedemia, ischemic heart disease, and claudication or rest pain in the lower limbs, was taken and recorded. Detailed systemic examination along with palpation for diminution or absence of dorsalis pedis and posterior tibial pulses in both the limbs was performed. Hypertension was considered according to the JNC VII criteria from stage-1 onward. EKG was completed for every patient. Systolic blood pressure was recorded in the supine position starting with the right arm, the right leg, the left leg, and the left arm by using an OMRAN Digital Automatic Blood Pressure Monitor (Model SEM-1, HEM-7051-C 12). Blood pressure was repeated in the limbs, whenever there was an error recorded on the apparatus. The ABI was calculated by dividing the ankle systolic blood pressure by the brachial systolic blood pressure of the respective sides.

Whenever the ABI was below 0.9, it was considered as a positive test according to the established criteria and such patients were subjected to color Doppler evaluation of both the lower limbs. Those patients who had positive ABI that was confirmed by Doppler were taken as true positives, and those who had a positive test and negative Doppler were taken as false positive. An equal number of age- and sex-matched patients from the enrolled patients, who showed a negative test, were subjected to Doppler evaluation; those who showed obstruction were taken as false negatives and those who did not were taken as true negatives. A lipid profile was performed for all patients who were ABI positive as well as the controls at a standard laboratory (NABL recognized). The TC/HDL ratio of > 4 was taken as the standard for considering hyperlipidemia. All the findings were computerized and analyzed. Statistical analyses were performed by using the t-test, ×2 test, and proportionate tests. Sensitivity and Specificity of the ABI were calculated by this method.

## Results

There were 120 males (60.0%) and 80 females (40.0%) with a mean age of 60.1 ± 9.5 and 61.5 ± 11 years, respectively [Table 1]. Analysis of the data revealed that 84.0% of the males and 81.2% of the females had diabetes from six to 20 years. Hypertension was the most common risk factor in both sexes, and smoking was confined to males and was also not high. Ischemic heart disease (IHD) was present in a quarter of the patients. Symptoms suggestive of PAD were present in < 25% and signs of vascular obstruction in the form of weak or absent peripheral pulses were seen in only 33% of the males and 42% of the females, respectively. The ABI test was positive in 12 males (10%) and six females (7.5%) [Table 2]. Eleven males (91.6%) had a true positive test, whereas, it was three (50.0%) in females. A true negative test was also higher in males when compared to females (nine vs. three). Duration of diabetes, hypertension, IHD, and signs and symptoms of PAD were all significantly higher among males who had true positive tests when compared to those who had a true negative test. This was also the case in females, except that IHD was seen in higher proportions among true negatives. Dyslipedemia was not seen in females who showed a positive ABI as well as in ABI negative controls [Table 3]. The ABI calculated by this method had a Sensitivity of 70.0% and a Specificity of 75.0%. A qualified statistician analyzed all the figures.

**Table 1:** Age and Sex Distribution

Age group (years)	Males (N-120) (%)	Females (N-80) (%)
< 40	3(2.5)	3(3.8)
40 – 49	13(10.8)	7(8.8)
50 – 59	44(36.6)	30(37.5)
60 – 69	40(33.3)	21(26.2)
70 – 79	17(14.1)	14(17.5)
> 80	3(2.5)	5(6.2)
Total	120(100)	80(100)
Mean ± SD	60.1 ± 9.5	61.5 ± 11

**Table 2:** Result of ABI evaluations by the Oscillometric method

Results	Number	ABI-Positive	%
Males	120	12	10.0
Females	80	06	7.5
Total	200	18	9.5

**Table 3:** Analysis of True positives and True Negatives

Parameters	True positives		True Negatives	
		
	M- (N&%)	F- (N&%)	M- (N&%)	F- (N&%)
Age (Av-years)	69	68.5	68.5	70.5
DM (11 – 15)	9 (81.5)	3 (100)	7(77)	2(66)
BMI (Av)	26.7	24.8	27.0	24.3
Smoking	5(45)	0 (0)	3(33)	0.0
Hypertension	10(90)	3 (100)	7(78)	1(33)
Dyslipedemia	5(45)	0(0.0)	5(55)	0(0)
IHD	4(36)	1(33)	2(22)	2(66)
Symptoms	6(55)	1(33)	1(11)	0(0)
Signs	9(81)	3(100)	2(22)	1(33)

## Discussion

The present study had two main objectives. The first one was to know whether the oscillometric method used for the evaluation of ABI in this study was good enough to be used in general practice by clinicians, to detect a larger number of PAD cases. The second one was to correlate the signs and symptoms with PAD after ABI. The ancillary objective was to look at the importance of the risk factors.

Among the number of tests that are available to detect PAD, Ankle Brachial Pressure Index (ABI) is the method of choice that can be used in Outpatient situations. ABI measured using a handheld Doppler of 5 – 10 MHZ and the routine BP apparatus cuff — where both brachial systolic pressure and ankle systolic pressure are measured for dorsalis pedis and posterior tibial arteries of each lower limb — is considered as the gold standard measurement. It has a sensitivity of 89%, specificity of 99%, PPV of 90%, and NPV of 99%, with an overall accuracy of 98%. ABI of < 0.9 showing possibility of PAD, <0.8- being highly likely, 0.5 – 0.8- depicting single segment occlusion, and <0.5 suggestive of multi-segment disease[1,20,23,29]

In spite of such a good test available, few general physicians measure the ABI in their daily practice, for several reasons. The device may not be available to them, as it is expensive. Yet even if the device is available, few of them use it, as it takes a lot of their time to perform the test and training is required for the person who uses the device.[10,9] Hence, a test that is automated, easy to perform, takes less time, and is less reliant on the specialized skills may facilitate measurement of ABI more frequently. Oscillometric (automated) determination of blood pressure is approved and is commonly available, reliable, and simple to use, This method has a sensitivity of 88%, specificity of 85%, PPV of 65%, and NPV of 96% (LLL) and 75, 95, 85, and 88%, respectively (RLL).[13] The present study that has used this method for ABI had a sensitivity of 70% and specificity of 75% as a whole.

There are controversies regarding the variability of ankle brachial systolic pressure. One study revealed that a single measurement is suitable for most epidemiological studies of athersclerotic PAD,[6] whereas, the ARIC study[11] suggested repeated measurements, as the reliability increased with repeat measurements[8] This study used single measurement in all the four limbs and whether the results would have been higher with repeated measurements is not known, but that could form the basis for another study.

There are also different opinions regarding the accuracy of the oscillometric device in measuring a-b pressure. One study[7] stated that this method played no role at all. Other studies have shown good results with the use of this device.[10,17]

It has been shown that the presence of claudication or the decrease of peripheral pulsations suffer from insensitivity.[23,26] It has also been stated that classical intermittent claudication can be as low as 11%.[14,15] The DP and PT can be absent congenitally in 8% and 2% of the cases.[12] This study shows that the symptoms of PAD are far lower both in males and females, and signs of decrease or absence of pulsations in either DP or PT, in one or both limbs, was higher when compared to the symptoms. This high figure may possibly be because the study is in diabetics, who are more prone to develop atherosclerotic vascular disease when compared to the general population, however, all of them did not show a positive ABI. What was important was the fact that in those who were ABI positive, the symptoms that were present were much higher in both males and females, whereas, it was very low in males; and females in whom the ABI was negative had no symptoms. So also was the fact that signs were present in most of the males and all females who were true positive, whereas, they were meager in males and females who were true negative [Table 3]. This shows that signs are important despite the absence of symptoms, and peripheral pulsations should be sought in all clinical examinations, which will pave a way to perform the ABI. Even in those six patients who were false negative, three patients had symptoms and three patients had signs. The ABI in five out of the ten limbs was between 0.92 and 0.97, and in one it was 1.35, which was hyper normal, which is common in diabetics with medial sclerosis.[13,16,29] This probably shows that if the upper limit of ABI for abnormality is raised from < 0.9 to < 1.0, probably more cases would be found, as clinicians tend to ignore the symptoms if the ABI is >0.9.

The confirmation of PAD was done on patients who had shown positive ABI by Arterial Duplex Ultrasound, because of the fact that this method is a precise method for defining obstruction and stenosis, as it has a sensitivity of 95% for occlusion and 92% for stenosis and specificity of 99 and 97% for occlusion and stenosis, respectively.[4]

As far as the risk factors are concerned, diabetes and smoking are the strongest risk factors and other well-known risk factors are advanced age, hypertension, and dyslipedemia.[18,21,23,24,25,27] This study, as it was done in diabetics, had the strongest risk factor. The mean duration of diabetes was 11.2 ± 5 years for males and 10.8 ± 5.5 years for females. Hypertension emerged as the most important risk factor in all the participants in general and was present in most of the males and all the females who were true positive [Tables 3]. Ischemic heart disease was present in a third of the true positives, which was significant, and conveyed the importance of detecting PAD as it was associated with CHD more often and could be a forerunner for it.[5,28] It also showed that advanced diabetic age was also very important as most of the males and all the females who were confirmed with having the PAD had the disease from 11 to 20 years, except one, who was a smoker, had PAD, and then developed diabetes. Another patient who was false negative had symptoms and signs and yet had a normal ABI, as the obstruction was at a higher level [Table 3].

This study has shown that the oscillomeric method of ABI is most useful in males. True positivity and true negativity are higher in males when compared to females, in spite of females having similar risk factors, which is difficult to explain.

The oscillometric method of elicitation of ABI has its limitations. The apparatus has to be a standard one. Individual BP on DP and PT cannot be performed, as it is performed by the Doppler; hence the systolic blood pressure elicited in the lower limb is an average of pressures in each artery. Obstruction in a particular vessel cannot be distinguished, as the individual ABI for that vessel cannot be performed. Similarly, ABI can be normal if the obstruction is higher. The sensitivity, specificity, PPV, and NPV are not as high as in the standard method.

In spite of these limitations, this method scores over the standard method, by its simplicity, no bias as far as readings are concerned, as they are automatic, negligible cost of the instrument (Rs. 3000), ease of performance, and the rapidity with which it can be done, (six to eight minutes) not only by the doctors, but even the paramedical staff. Confirmation has to be completed with the Doppler in either of the methods. When that is so, the oscillometric method can be used on a daily basis as an outpatient procedure, to detect a larger number of PAD cases.

## Conclusions

This study has proved that the oscillometric method of ABI is a very useful procedure in spite of its limitations. By repeating the test, the sensitivity and specificity may probably increase. What is important is the fact that this test can be performed by primary care physicians even in their primitive set up. Even as detecting more number of PAD cases is the primary objective, this method scores over other methods. More studies on a larger number of cases using this method would pave the way for standardization of the method. We recommend that all the doctors to try this method and evaluate the results.

## References

[1] Orchard TJ, Strandness DE (1993). Assessment of peripheral vascular disease in diabetes. Report and recommendations of an international workshop sponsored by the American Diabetes Association and the American Heart Association September 18-20, 1992 New Orleans, Louisiana. Circulation.

[2] Powers KB, Vacek JL, Lee S (1999). Noninvasive approaches to peripheral vascular disease. Postgrad Med.

[3] Sacks D, Bakal CW, Beatty PT, Becker GJ, Cardella JF, Raabe RD (2003). Position Statement on the use of ABI in the evaluation of patients with peripheral vascular disease, A Consensus statement developed by the standards division of society of Interventional Radiology. J Vasc Interv Radiol.

[4] Mohler ER (2003). Peripheral Arterial Disease- Identification and Implications. Arch Intern Med.

[5] Beckman JA, Higgins CO, Gerhard-Herman M (2006). Automated oscillometric determination of ankle-brachial index provides accuracy necessary for office practice. Hypertension.

[6] (2004). Peripheral Arterial Disease in Diabetes. Diabetes Cadiovasc Dis Rev.

[7] Mayfield JA, Reiber GE, Maynard C, Czerniecki J, Sangeorzan B (2004). The Epidemiology of lower extremity disease in veterans with Diabetes. Diabetes Care.

[8] Stoffers HE, Kester AD, Kaiser V, Rinkens PE, Knottnerus JA (1997). Diagnostic value of signs and symptoms associated with peripheral arterial disease seen in gen practice: A Multivariable Approach. Med Decis Making.

[9] Marso SP, Hiatt WR (2006). Peripheral arterial disease in patients with diabetes. J Am Coll Cardiol.

[10] Jaff MR (2002). Lower extremity arterial disease, Diagnostic aspects. Cardiol Clin.

[11] Leng GC, Fowkes FG, Lee AJ, Dunbar J, Housley E, Ruckley CV (1996). Use of ankle brachial index to predict CV events and death, A cohort study. BMJ.

[12] Feigelson HS, Criqui MH, Fronek A, Langer RD, Molgaard CA (1994). Screening for peripheral arterial disease, the sensitivity, specificity and predictive value of non invasive tests in defined population. Am J Epidemiol.

[13] American Diabetes Association^®^ Inc 2004 (2004). Consensus Statement. Peripheral Arterial disease in people with Diabetes. Clin Diabetes.

[14] Ramakrishna P, Tripathy BB (2008). Peripheral Arterial Disease in Diabetes. RSSDI Text book of Diabetes Mellitus.

[15] Ray SA, Srodon PD, Taylor RS, Dormandy JA (2005). Reliability of ankle: Brachial pressure index measurement by junior doctors. Br J Surg.

[16] Mathew JG, Pickup JC, Williams G (2002). Foot problems in diabetes. Text Book of Diabetes.

[17] Fowkes FG, Housley E, Macintyre CC, Prescott RJ, Ruckley CV (1988). Variability of ankle-brachial systolic pressures in the measurement of athersclerotic peripheral arterial disease. J Epidemiol Community Health.

[18] Weatherley BD, Chambless LE, Heiss G, Catellier DJ, Ellison CR (2006). The reliability of the ankle-brachial index in the Atherosclerosis Risk In Communities (ARIC) study and the NHLBI Family Heart Study (FHS). BMC Cardiovasc Disord.

[19] Stoffers HE, Kester AD, Kaiser V, Rinkens PE, Kitslaar PJ, Knottnerus JA (1996). The diagnostic value of the measurement of the ankle-brachial systolic pressure index in primary health care. J Clin Epidemiol.

[20] Ramanathan A, Conaghan PJ, Jenkinson AD, Bishop CR (2003). Comparison of ankle-brachial pressure index measurements using an automated oscillometric device with standard Doppler ultrasound technique. ANZ J Surg.

[21] Lee CW, Park KY, Son S, Kim IJ, Kim YK (2003). Usefulness of ankle brachial pressure index measured using PhotoPlethysmography and Automated BP measurement device. Korean J Med.

[22] Corpus RA, Marso SP, Stern DM (2004). Diabetes and Peripheral Artery Disease. Diabetes and Cardiovascular Disease.

[23] Creager MA, Loscalzo J (2008). Vascular diseases of extremities. Harrisons Principles of Internal Medicine.

[24] Aulivola B, Hamdan AD, LoGerfo FW, Johnstone MT, Veves A (2005). Peripheral vascular disease in patients with Diabetes Mellitus. Diabetes and cardiovascular disease.

[25] Meijer WT, Grobbee DE, Hunink MG, Hofman A, Hoes AW (2000). Determinants of Peripheral Arterial Disease in the Elderly: The Rotterdam Study. Arch Intern Med.

[26] Adler AI, Stevens RJ, Neil A, Stratton IM, Boulton AJ, Holman RR (2002). UKPDS 59, hyperglycemia and other potentially modifiable risk factors for peripheral vascular disease in Type2 Diabetes. Diabetes Care.

[27] Sheehan P (2004). Peripheral arterial disease in people with diabetes: Consensus statement recommends screening. Clin Diabetes.

[28] Leibson CL, Ransom JE, Olson W, Zimmerman BR, O'fallon WM, Palumbo PJ (2004). Peripheral arterial disease, diabetes and mortality. Diabetes Care.

[29] Clark LT, Baweja P, Uzodinma R, Clark LT (2007). Coronary risk equivalence. Cardiovascular Disease and Diabetes.

